# Qualitative Invariant Effects Arise from Neural Constraints: Common Architecture and Sources of Individual Differences

**DOI:** 10.5334/joc.175

**Published:** 2021-08-27

**Authors:** Andrea Stocco

**Affiliations:** 1 Department of Psychology, University of Washington, Seattle, WA 98195, US

**Keywords:** Mathematical modelling, Neuropsychology, fMRI

## Abstract

The distinction between qualitative and quantitative effects can be meaningfully related to the distinction between “architecture” and “model” in computational cognitive science. In turn, this distinction can be related to differences between the invariant systems-level organization of the human brain and individual differences in structural and functional activity. Taken together, this presents an iterative new way to answer Newell’s “20 Questions” problem and to systematize psychological effects as belonging to either architecture or the individual variations within it. Although some limits to this approach exist (for example, Individual differences in strategy might affect whether an effect is qualitative or quantitative), the approach might also shed light on how to account for special populations (neurological or psychological patients) within the same framework.

Psychologists have always used behavioral tests to make inferences about the brain and, although it has been most commonly associated with psychometrics, the study of individual differences can be used for this aim as well. But how should individual differences be measured? As Haaf and Rouder point out in the main article, raw behavioral data is noisy and contaminated by errors. Because of this, it is becoming more and more common to use sophisticated computational models to measure individual differences, and then use the parameters of these models to analyze neural data. Such models include, for example, decay models of memory (Sense et al., 2016), drift-diffusion models (White et al., 2014), and reinforcement learning models (Collins, 2018). In such an approach, “qualitative” differences do not really occur; individual differences show up as different parameter values, and differences between individuals are all “quantitative” in nature. This modeling approach, however, performs a sort of sleight of hand, making the qualitative differences disappear by imposing constraints on behavior through the equations they are based upon. This raises another problem: how do we know which model is correct? For most phenomena, multiple possible modeling approaches exist. For example, intrusive fearful memories in Post-Traumatic Stress Disorder have been modeled through reinforcement learning (Myers et al. 2013) or through differently decaying memory traces (Smith et al., in press). These models provide very different and incompatible worldviews of the mechanisms underlying this, and it would be desirable to adjudicate between them before analyzing quantitative differences between individuals.

In many ways, this problem is analogous to the one singled out by Newell (1973) in his “You can’t play twenty questions with nature and win” paper. Newell argued that, instead of developing problem-specific models, different phenomena should be modeled within the same, invariant “cognitive architecture”. However, exactly as the same behavior can be explained by modeling frameworks, it can also be accounted for at the level of the architecture or at the level of the specific model.

The work by Haaf and Rouder provides a useful way out of this impasse. Almost by definition, a qualitatively invariant effect must be part of the architecture, as it must be due to processing constraints that are shared across all humans. In other words, each qualitatively invariant effect that exists represents a constraint that any architecture must respect. Cognitive architectures could even be compared on the basis of the number of qualitatively invariant effects they predict. Once an architecture is established, quantitative differences between individuals might still be interpreted as reflecting individual differences in specific architecture parameters.

## Qualitatively Invariant Effects as Brain Constraints

Although Newell was specifically addressing the architecture of the mind, the problem can be easily extended to understanding the brain—after all, the mantra of biology is that function is determined by structure, and the structure that supports cognition is the brain. In this sense, the architecture can be conceived as the large-scale organization of pathways that connect different brain regions which are functionally specialized. These pathways pose fundamental constraints on how information flows and must pass through the brain and, although our brains are different, they also share a common organization. Indeed, recently, we have been looking precisely at whether this common organization of pathways can be identified in the human brain, using neuroimaging data collected in the Human Connectome Project. The results show that such a high-level, common organization can be identified (Stocco et al., 2021) and that it closely reflects the main tenet that most cognitive architectures have converged upon (Laird et al., 2017). Such a common organization can be interpreted as the source of invariant cognitive effects across individuals.

As Haaf and Rouder point out, many psychologists believe that truly invariant effects might not exist and that every behavioral effect must exhibit some form of qualitative difference. The architectural view, however, provides a framework to interpret rare and exceptional qualitative differences. Although an architecture should be invariant across individuals, some exceptions can be easily thought of. For example, patients suffering from brain damage or neurological disease can be expected to have such a high degree of neural diversity that they can be legitimately claimed to have a different architecture. Consider, for example, an extreme case such as patient HM (Corkin 2002). Due to bilateral removal of the hippocampus, HM was unable to form new memories. His post-surgical behavior cannot be interpreted on a quantitative scale of, for instance extreme memory decay (Zhou et al. 2021); instead, it is consistent with what would be expected if we take any cognitive architecture and selectively remove one specific module.

## Summary

In summary, the distinction between qualitative and quantitative effects can be put into correspondence with the distinction between a common architecture and individually parametrized models developed within it. Exactly like quantitative differences in model parameters have been used to interpret individuals differences in neural data (Zhou et al. 2021), qualitative effects might provide the key to refine our understanding of the cognitive architecture and the fundamental organization of the human brain.
